# Large-scale discovery of previously undetected microRNAs specific to human liver

**DOI:** 10.1186/s40246-018-0148-4

**Published:** 2018-03-27

**Authors:** Brenda C. Minatel, Victor D. Martinez, Kevin W. Ng, Adam P. Sage, Tomas Tokar, Erin A. Marshall, Christine Anderson, Katey S. S. Enfield, Greg L. Stewart, Patricia P. Reis, Igor Jurisica, Wan L. Lam

**Affiliations:** 1 Department of Integrative Oncology, British Columbia Cancer Research Centre, Vancouver, BC Canada; 2 Krembil Research Institute, University Health Network, Toronto, ON Canada; 3 Faculty of Medicine, São Paulo State University (UNESP), Botucatu, SP Brazil

**Keywords:** Liver, Non-coding RNA, Novel miRNA, Tissue specificity, Liver cancer

## Abstract

MicroRNAs (miRNAs) are crucial regulators of gene expression in normal development and cellular homeostasis. While miRNA repositories contain thousands of unique sequences, they primarily contain molecules that are conserved across several tissues, largely excluding lineage and tissue-specific miRNAs. By analyzing small non-coding RNA sequencing data for abundance and secondary RNA structure, we discovered 103 miRNA candidates previously undescribed in liver tissue. While expression of some of these unannotated sequences is restricted to non-malignant tissue, downregulation of most of the sequences was detected in liver tumors, indicating their importance in the maintenance of liver homeostasis. Furthermore, target prediction revealed the involvement of the unannotated miRNA candidates in fatty-acid metabolism and tissue regeneration, which are key pathways in liver biology. Here, we provide a comprehensive analysis of the undiscovered liver miRNA transcriptome, providing new resources for a deeper exploration of organ-specific biology and disease.

**Electronic supplementary material:**

The online version of this article (10.1186/s40246-018-0148-4) contains supplementary material, which is available to authorized users.

MicroRNAs (miRNAs) are known to promote post-transcriptional fine-tuning of gene expression through complementary binding to target mRNA sequences [1]. Their wide-reaching effects are attributed to the fact that a single miRNA can target dozens to hundreds of genes, often affecting multiple nodes of a given signaling pathway [1]. In the liver, miRNAs are believed to orchestrate cell lineage differentiation during organ development, the modulation of homeostatic liver functions such as cholesterol and lipid metabolism, and disease [2, 3]. Clinically, miRNAs hold prognostic and therapeutic value both as biomarkers and therapeutic targets. For example, Miravirsen is a miR-122 antagonist emerging as a promising treatment for hepatitis C infection, which has progressed through Phase 2a clinical trials [4].

Initial attempts to characterize the human miRNA transcriptome were mostly limited to the discovery of abundant miRNA sequences and/or sequences that are conserved across several tissue types. This restriction may preclude miRNA transcripts with expression patterns that are more specialized to individual tissues or cell lineages [5, 6]. Indeed, recent genome-wide studies using next-generation sequencing have suggested the existence of human-specific previously undetected miRNAs, and they have been shown to exhibit high tissue specificity [5–8]. Therefore, the discovery of such miRNA sequences may uncover novel tissue-specific regulatory mechanisms relevant to developmental biology and disease pathology. In this study, we performed a large-scale discovery of miRNA candidates previously undescribed in liver tissue and showed that these sequences exhibit tissue-specific expression patterns, as well as involvement in liver biology and disease.

Non-malignant liver small RNA sequence data was obtained from The Cancer Genome Atlas (TCGA; *n* = 47). Previously unannotated miRNA sequence discovery was performed using the miRDeep2 algorithm, which scans the transcriptome for novel miRNA candidates and compares them with known miRNA sequences available in public databases, such as miRBase [9]. This established miRNA detection algorithm uses a statistical model to measure the likelihood of a detected small RNA sequence to be a putative novel miRNA. Primarily, this model assesses the hairpin structure of the predicted miRNA precursor and recognizes whether the precursor gives rise to the three products of miRNA processing by DICER, namely (i) mature miRNA, (ii) star sequence, and (iii) hairpin loop [9]. The likelihood of a detected small RNA sequence to be a true positive hit is reflected in the miRDeep2 score [9]. However, the selection of true positives based solely on the provided miRDeep2 score may still yield a large amount of false positive candidates [7]. To overcome these limitations, we applied several additional filtering steps to reduce the rate of false positives.

The initial miRDeep2 analysis discovered 263 unannotated miRNA candidate sequences. First, this output was filtered by the number of reads corresponding to the mature sequence (≥ 10), a significant (*p* ≤ 0.05) probability of a hairpin-like secondary structure, sequence similarity with annotated miRNAs in the miRBase repository, and a miRDeep2 score ≥ 1, yielding a set of 110 candidate unannotated miRNA sequences (Fig. 1). We further assessed the similarity of these newly detected miRNAs with annotated miRNAs using the novoMiRank tool [7], which provides z-scores to each sequence based on 24 different features. Briefly, higher z-score numbers indicate less similarity to known miRNAs. Thus, while reads of these sequences may still be detected, miRNAs assigned a z-score ≥ 1 have an increased probability of representing false-positive candidates (Additional file 1: Table S1). Finally, we removed any predicted miRNA sequence with a GC-content ± 2 STD from the mean of currently annotated sequences (Additional file 2: Figure S1). Collectively, our filtering criteria resulted in the identification of 103 unique unannotated miRNA candidates, representing a substantial increase in the total number of miRNAs expressed in human liver (Fig. 1 and Additional file 1: Table S1). Additionally, these miRNA candidates were found to have similar sequence composition, folding structures and genomic distribution relative to annotated miRNAs, further supporting their identity as true positive miRNA sequences (Fig. 2).

**Fig. 1:**
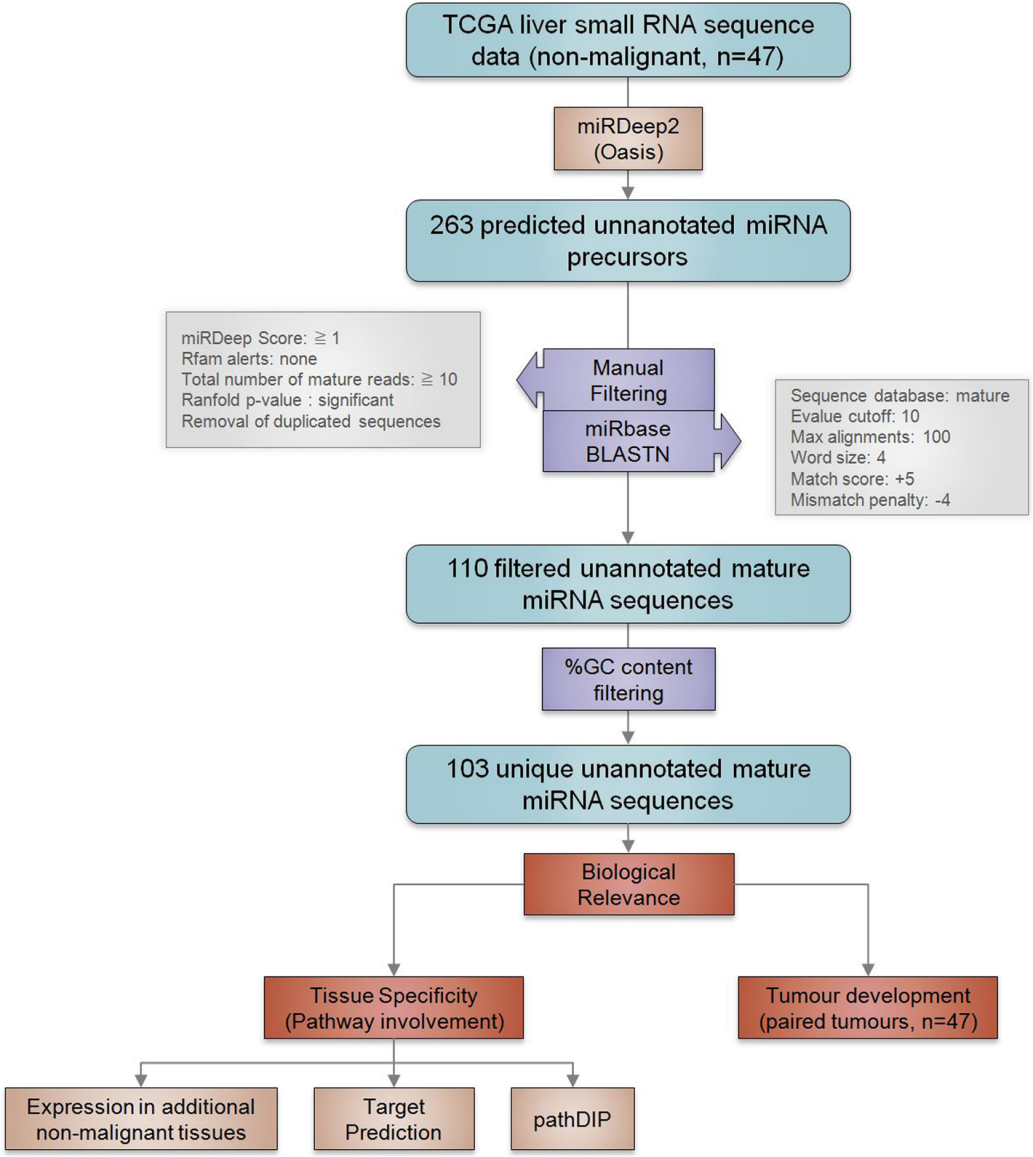
Analysis flow diagram. Detailed description of the analysis pipeline applied for the discovery of unannotated miRNA candidates in the liver and investigation of their possible biological functions

**Fig. 2:**
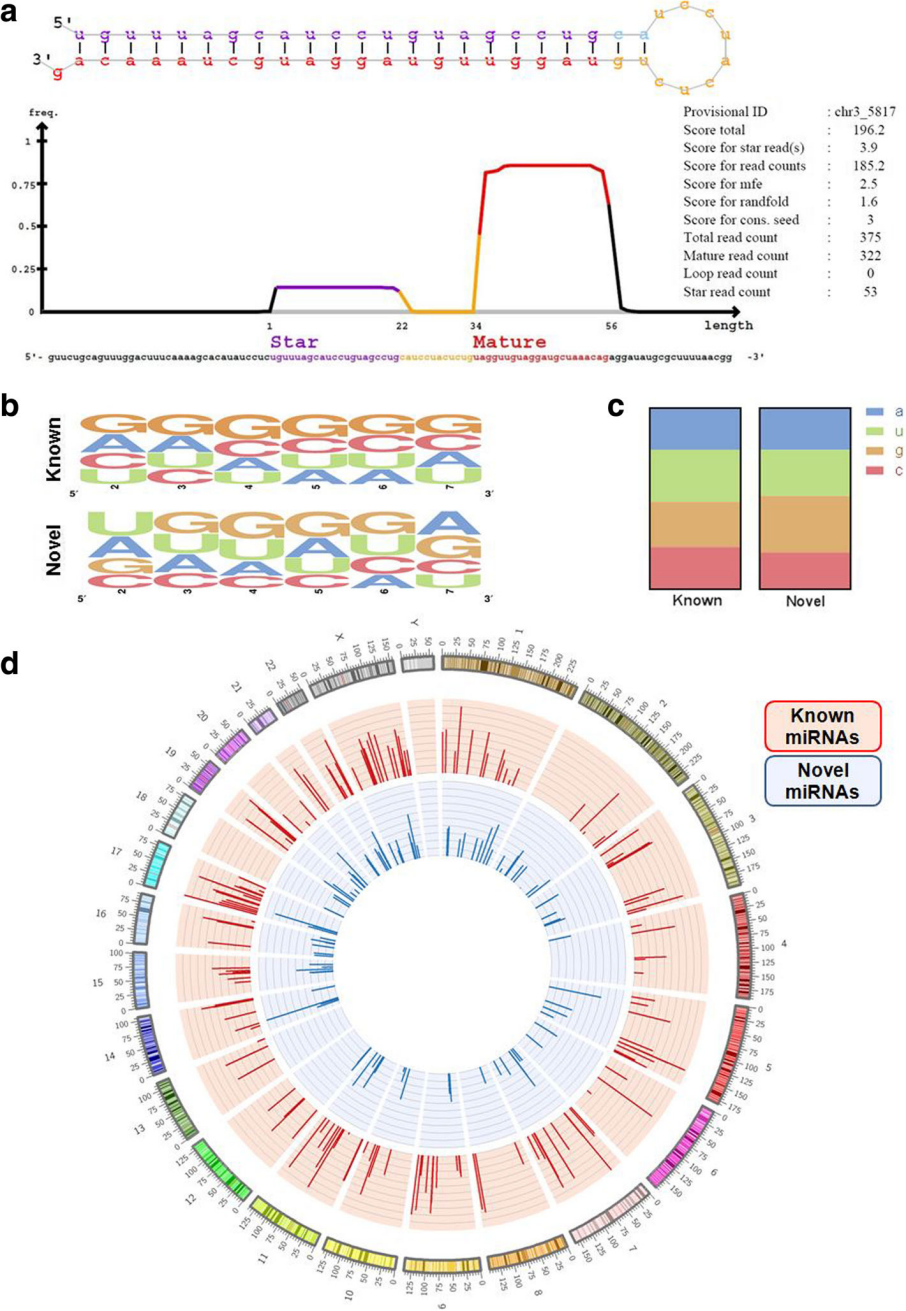
Comparison of annotated and unannotated miRNAs expressed by liver samples. **a** Detailed output from the miRDeep2 algorithm demonstrates that the unannotated miRNA candidates discovered display miRNA-like folding structures. **b** Sequence logo representation of average nucleotide composition in each position of the seed regions of annotated and unannotated miRNAs. **c** Average nucleotide composition in all positions of annotated and unannotated miRNAs. **d** Circos plot representation of the genomic localization of unannotated relative to annotated microRNAs

Next, to determine the tissue specificity of these miRNA transcripts, the expression of the 103 previously unannotated miRNA candidates was queried in small RNA sequencing data derived from organ sites representing distinct anatomical regions and that differ in germ layer derivation (endoderm or mesoderm). The tissues investigated were the pancreas (*n* = 4), bile duct (*n* = 9), head and neck (*n* = 42), stomach (*n* = 45), kidney (*n* = 71), and lung (*n* = 91). We performed non-linear t-Distributed Stochastic Neighbor Embedding (t-SNE) dimensionality reduction on the normalized expression levels of the 103 unannotated miRNA transcripts against the aforementioned tissues. The expression pattern of these miRNA sequences was similar in both the liver and bile duct, corroborating their shared developmental lineage. In contrast, their expression in the liver is clearly distinct from the head and neck, stomach, kidney, and lung samples (Fig. 3), suggesting that our unannotated miRNA candidates have a unique pattern of expression that relies on cell lineage and that they may be relevant to liver-specific biology.

**Fig. 3:**
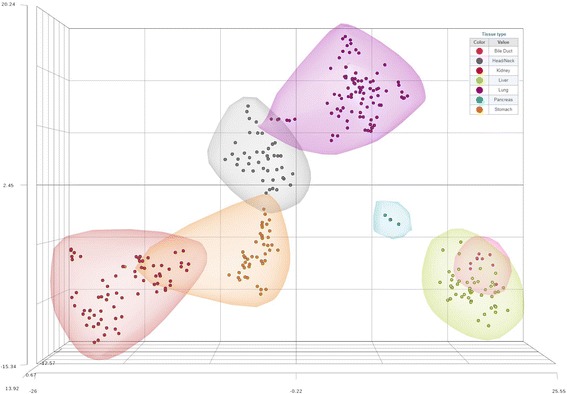
Tissue-specific expression patterns of the unannotated miRNA transcripts. t-Distributed Stochastic Neighbor Embedding (t-SNE) analysis of non-malignant tissues from The Cancer Genome Atlas: liver (*n* = 47), pancreas (*n* = 4), bile duct (*n* = 9), head and neck (*n* = 42), kidney (*n* = 71), lung (*n* = 91), and stomach (*n* = 45). The analysis was performed using normalized expression levels derived from the loci encoding the 103 unannotated miRNA candidates identified in the liver

To identify the pathways regulated by the unannotated miRNAs, we analyzed their predicted targets. We restricted our analysis to protein-coding genes that were identified as targets by at least two of the three algorithms used and were predicted to be targeted by at least 10% of our novel miRNA sequences (Additional file 3: Figure S2). From this, we identified a total of 723 protein-coding gene targets of the newly detected miRNA candidates in the liver.

Strikingly, subsequent pathway enrichment analysis revealed that the 723 predicted targets are enriched (*p* < 0.001) in pathways that are important to normal and diseased liver biology (Fig. 4). These pathways include the following: fibroblast growth factor receptor (FGFR) signaling pathways, epidermal growth factor receptor (EGFR) signaling pathway, DNAX-activating protein of 12 kDa (DAP12) signaling, and granulocyte-macrophages colony-stimulating factor (GM-CSF) mediated signaling. In the liver, the FGFR pathway has been shown to modulate cholesterol and fatty acid metabolism and has been associated with chronic liver diseases and hepatocellular carcinoma (HCC) [10]. Likewise, the EGFR pathway plays a role in liver regeneration and is also associated with HCC aggressiveness through the activation of cells that secrete extracellular matrix components [11]. Lastly, the DAP12 and GM-CSF pathways participate in immune regulation and inflammatory response by modulating the maturation of hepatic dendritic cells and the formation of inflammatory granulomas, respectively [12, 13]. As these newly detected miRNA sequences are predicted to target key pathways in liver biology and disease, their discovery may be a cornerstone for identifying new regulatory mechanisms that may be disrupted in liver pathologies.

**Fig. 4:**
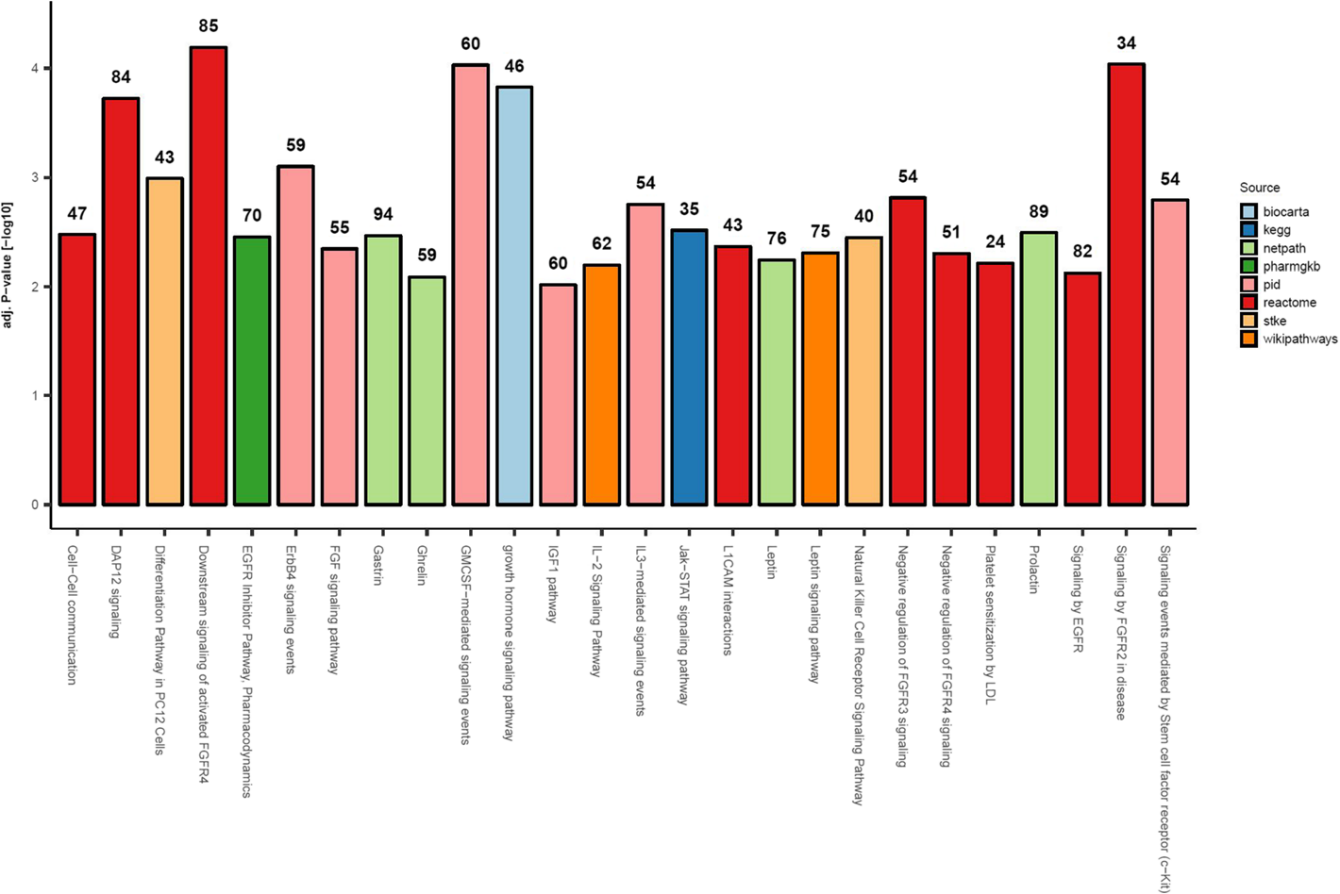
Biological relevance of the unannotated miRNA transcripts. Pathway enrichment analysis (pathDIP) of 723 genes that were predicted to be targeted by at least 10% of the newly detected miRNA candidates in the liver. Bar height indicates the FDR corrected enrichment *p* value with the number of target genes in that pathway denoted at the top

In order to further assess the biological relevance of the unannotated miRNA candidates, we sought to evaluate whether these sequences are deregulated in corresponding tumor samples. We compared the expression of the miRNAs between matched non-malignant and tumor tissues. Strikingly, 83 of the 103 miRNA sequences had lost (*n* = 65) or reduced (*n* = 18, Wilcoxon signed-rank test corrected *p* value < 0.05) expression in tumor samples (Additional file 4: Figure S3). Thus, the widespread decrease in expression of these unannotated miRNA sequences may contribute to liver tumorigenesis.

In conclusion, we have discovered 103 previously undetected miRNA candidates in the liver. Although further experimental validation is required to confirm these sequences, our results shed light into the existence of unexplored regulatory molecules in liver tissue. Most importantly, these unannotated miRNAs have not only a lineage-specific expression pattern but may also be regulators of key liver processes, including those relevant to pathogenesis. Collectively, our results have substantial implications for liver-specific miRNA biology, emphasizing the need to further explore the undescribed areas of the human transcriptome.

## Additional files

Additional file 1: **Table S1.** Output from miRDeep2 algorithm and novoMiRank scores for the 103 unique unannotated miRNA candidates. (XLS 59 kb)

Additional file 2: **Figure S1.** Percent GC content of unannotated and annotated miRNAs. Histogram plot of the percent GC content of the 110 filtered unannotated miRNAs predicted from miRDeep2 and all annotated miRNAs from miRBase v21. Dashed red lines indicate the two standard deviation thresholds from the mean of annotated miRNAs and were used as a filtering criteria. (JPEG 308 kb)

Additional file 3: **Figure S2.** Predicted targets and their overlaps across applied algorithms. A) Resulting number of predicted mRNA targets and their overlaps across the three different algorithms applied during target prediction analysis. B) Total number of predicted mRNA targets per unannotated miRNA. (JPEG 253 kb)

Additional file 4: **Figure S3.** Expression of the 38 unannotated miRNA transcripts in tumors. The expression of the 103 unannotated miRNAs was evaluated in a cohort of 47 liver tumor samples derived from the same patients in which the original miRNA prediction was performed. The expression of 38 miRNAs (39.1% of all the 103 miRNAs discovered) was detected in these tumor samples. (JPEG 167 kb)

Additional file 5: Detailed materials and methods. (DOC 109 kb)

## Abbreviations

DAP12: DNAX-activating protein of 12 kDa
EGFR: Epidermal growth factor receptor
FGFR: Fibroblast growth factor receptor
GM-CSF: Granulocyte-macrophages colony-stimulating factor
HCC: Hepatocellular carcinoma
miRNAs: MicroRNAs
TCGA: The Cancer Genome Atlas
t-SNE: t-Distributed Stochastic Neighbor Embedding
