# Supplementary figures and images for: Large-scale discovery of previously undetected microRNAs specific to human liver
Source: Hum Genomics. 2018 Mar 27;12:16. doi: 10.1186/s40246-018-0148-4 (PMC5870816; doi:10.1186/s40246-018-0148-4)

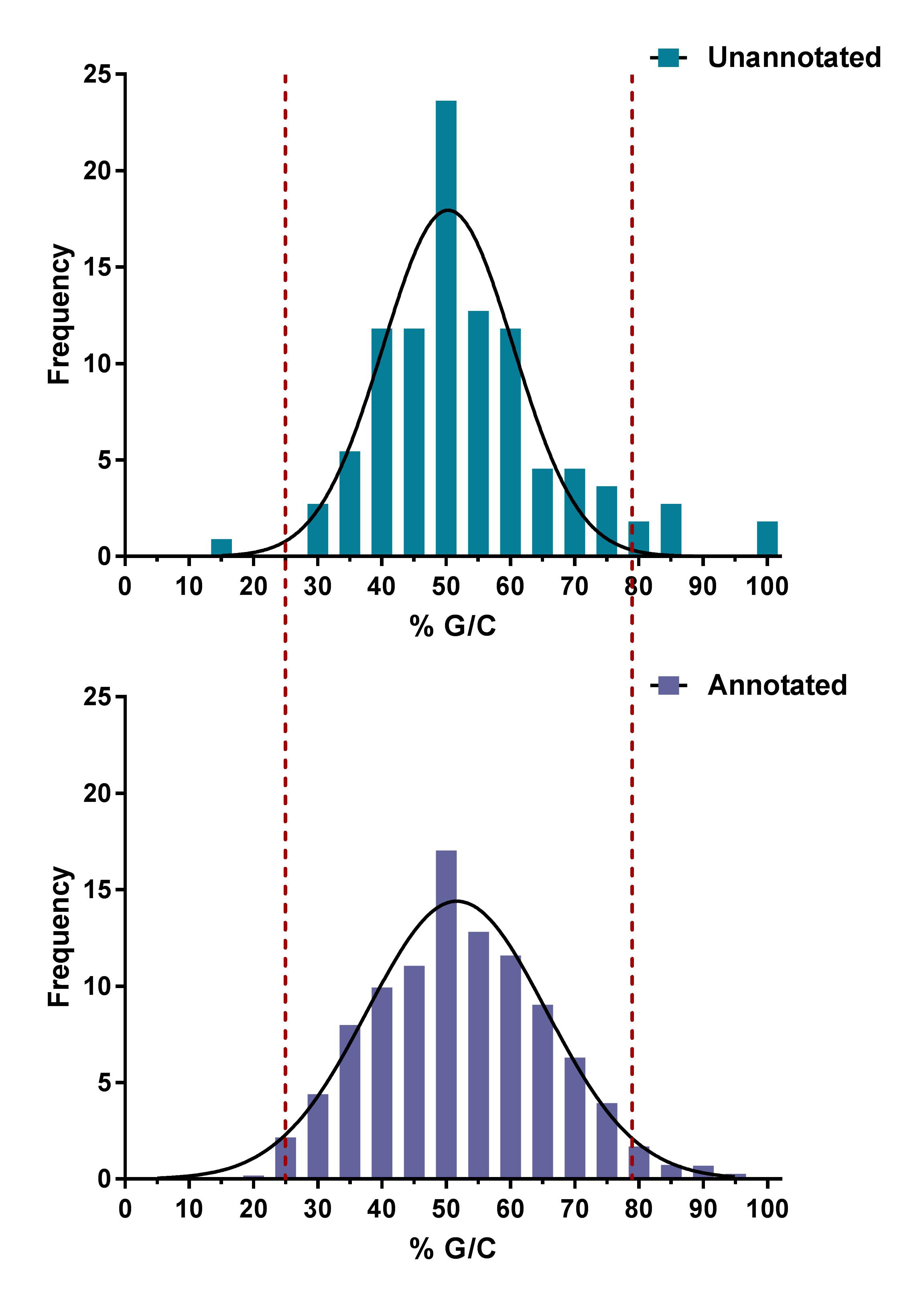

Supplement: Supplementary file 2 — Figure S1. Percent GC content of unannotated and annotated miRNAs. Histogram plot of the percent GC content of the 110 filtered unannotated miRNAs predicted from miRDeep2 and all annotated miRNAs from miRBase v21. Dashed red lines indicate the two standard deviation thresholds from the mean of annotated miRNAs and were used as a filtering criteria. (JPEG 308 kb) [file 40246_2018_148_MOESM2_ESM.jpg]

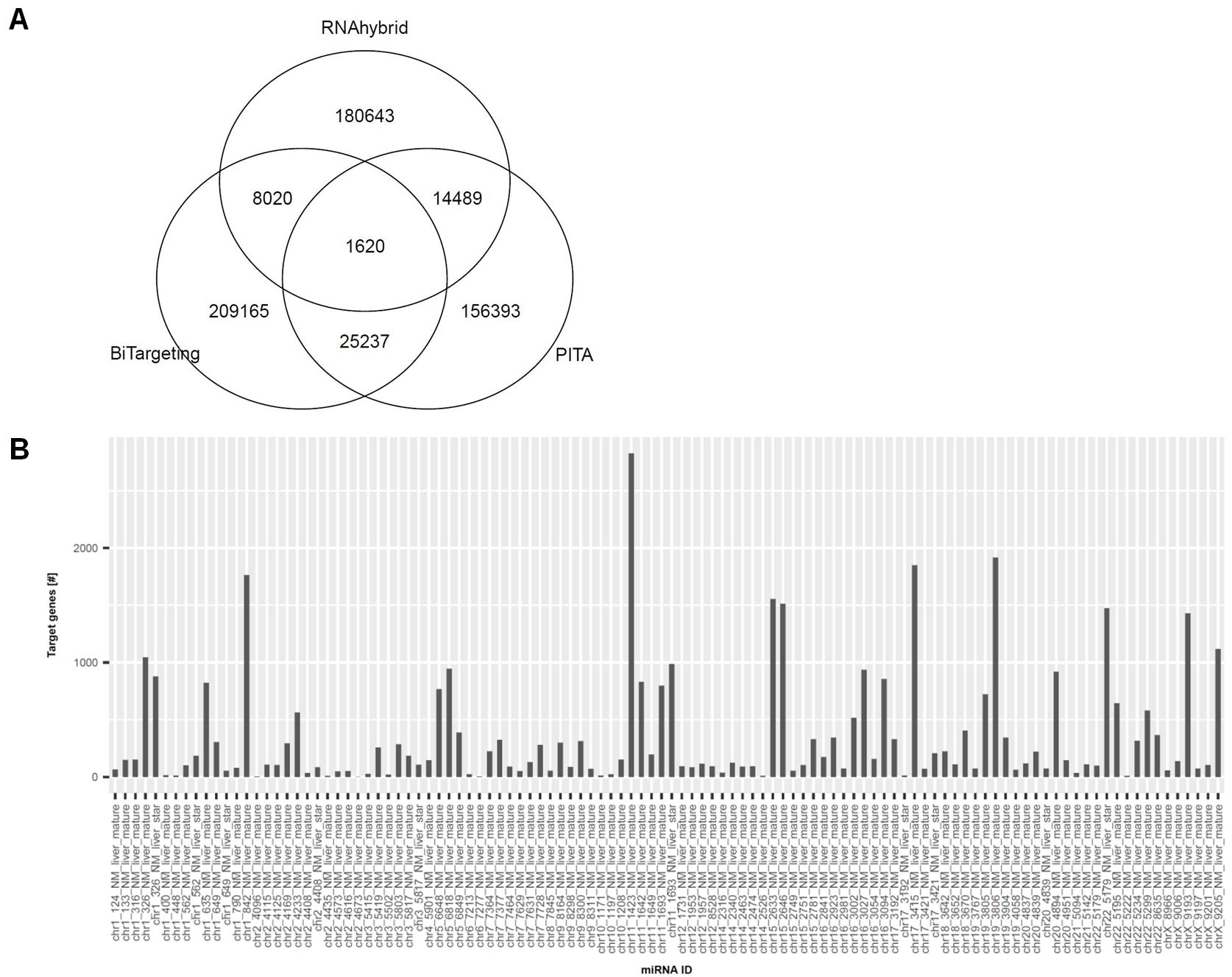

Supplement: Supplementary file 3 — Figure S2. Predicted targets and their overlaps across applied algorithms. A) Resulting number of predicted mRNA targets and their overlaps across the three different algorithms applied during target prediction analysis. B) Total number of predicted mRNA targets per unannotated miRNA. (JPEG 253 kb) [file 40246_2018_148_MOESM3_ESM.jpg]

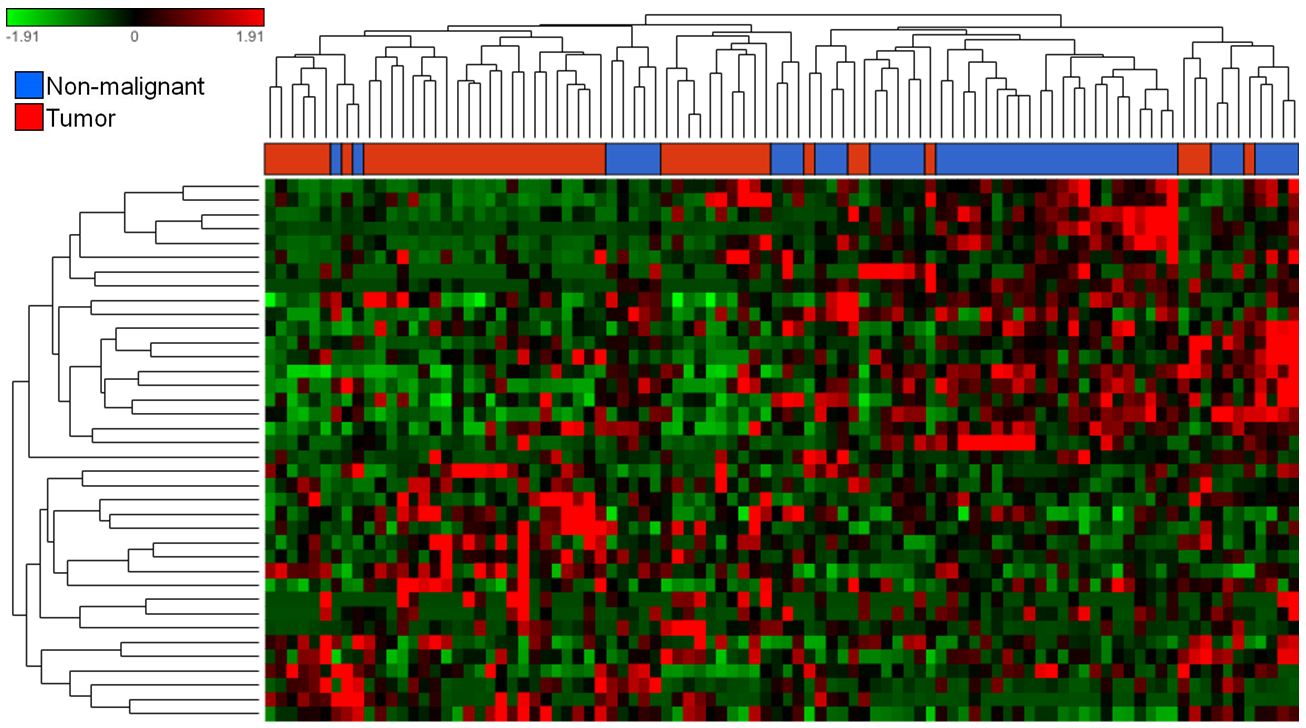

Supplement: Supplementary file 4 — Figure S3. Expression of the 38 unannotated miRNA transcripts in tumors. The expression of the 103 unannotated miRNAs was evaluated in a cohort of 47 liver tumor samples derived from the same patients in which the original miRNA prediction was performed. The expression of 38 miRNAs (39.1% of all the 103 miRNAs discovered) was detected in these tumor samples. (JPEG 167 kb) [file 40246_2018_148_MOESM4_ESM.jpg]
