# Supplementary material for: Large-scale discovery of previously undetected microRNAs specific to human liver
Source: Hum Genomics. 2018 Mar 27;12:16. doi: 10.1186/s40246-018-0148-4 (PMC5870816; doi:10.1186/s40246-018-0148-4)
Supplement: Supplementary file 5 — Detailed materials and methods. (DOC 109 kb) [file 40246_2018_148_MOESM5_ESM.doc]

**Supplemental Detailed Material and Methods**

**Data collection and small RNA sequencing**

A cohort of 47 paired non-malignant/tumor liver samples processed by The Cancer Genome Atlas (TCGA) Research Network (http://cancergenome.nih.gov/) were acquired from the Cancer Genomics Hub (cgHUB) Data Repository (dbgap Project ID: 6208). All small RNA sequencing data was generated using the Illumina HiSeq2000 platform.

**Pre-processing and known microRNA detection**

Small-RNA sequencing data was processed according to a previously published custom sequence analysis pipeline . Unaligned reads and quality scores (FASTQ format) were trimmed based on the Phred quality score (≥20). FASTQ files were then aligned to the human genome (hg38) using the Spliced Transcripts Alignment to a Reference (STAR) aligner. Annotated miRNA species were quantified based on loci from public databases . Chromosomal positions of expressed miRNAs were plotted against the hg38 karyotype obtained from UCSC Genome Browser. Annotated miRNA species were considered expressed if the reads across all samples summed to at least 10 reads.

**Detection of novel miRNA sequences and filtering parameters**

Prediction of unannotated miRNAs was performed through the Oasis platform using the miRDeep2 algorithm, which accounts for the relative free energy and random folding p-values of miRNAs (Supplemental Table 1). All predicted unannotated miRNA sequences were filtered based on i) the number of sequencing reads covering each locus, ii) no hits for rRNA/tRNAs, based on the Rfam database , and iii) predicted miRNA-like secondary structure (Figure 1). Additionally, all sequences were manually subjected through the miRBase BLASTN to ensure no sequence homology to known miRNAs annotated in miRBase v21 (<http://www.mirbase.org/search.shtml)>, as well as subjected through the miRCarta BLAST tool (https://mircarta.cs.uni-saarland.de/miblast/) and compared against four recent large-scale novel-miRNA discovery studies (Supplemental Table 1) . Only the sequences that showed an Expect (E) value >1 and/or at least one mismatch on the seed region (nt 2-7) in BLASTN against the miRBase repository were considered as unnanotated. The miRCarta repository collects miRNA predictions from recent publications and the results from miRMaster, which is a web-based tool for quantification of non-coding RNAs and prediction of novel miRNAs using the same miRDeep2 algorithm . The number of hits and the lowest E-value produced by the miRCarta BLAST are described in Supplemental Table 1. The presence of some of the sequences in miRCarta strengthens their candidacy as true miRNAs, as their prediction in additional tissue types suggests that they are less likely to be technical artefacts. Lastly, the distribution of the percent GC content of all sequences was also analyzed and compared against the distribution of annotated miRNAs to obtain a robust set of unannotated miRNA sequences. miRNA sequences that had a percent GC content outside two standard deviations of the mean were excluded from further analysis (Supplemental Figure 1). This robust set of unannotated miRNAs was then assessed using the web-based NovoMiRank tool to evaluate their similarity against annotated miRNAs of all miRBase versions (Supplemental Table 1) .

**miRNA Gene Target Prediction and Network Analysis**

Targets of the novel miRNAs were predicted by applying three distinct algorithms: BiTargeting (ver. May 2017) ; PITA v. 6.0 ; RNAhybrid (ver. May 2017) , using human genes 3’UTR sequences acquired from Ensembl through Biomart tool (https://www.ensembl.org/). All three prediction algorithms were executed using default parametrization. Obtained predictions were then reduced by filtering out predictions whose estimated binding energy was greater than -15.0 (BiTargeting), associated score greater than -10.0 (PITA), associated p-value was greater than 0.05 (RNAhybrid). Hereafter we considered only the miRNA-transcript associations which were confirmed by at least 2 out of 3 algorithms (Supplemental Figure 2A).

For each miRNA, we tested enrichment of its target genes across Gene ontology (GO) biological processes, KEGG pathways and Disease ontology instances. This was done using Bioconductor Package clusterProfiler . Finally, we selected 723 genes targeted by at least 10% of all 103 miRNAs, which were then subjected to comprehensive pathway enrichment analysis using pathDIP conducted across 15 distinct pathways resources (Extended pathway associations. Experimental plus orthologues plus FpClass -- High Confidence; Minimum confidence level for predicted associations: 0.99). Obtained results were visualized as a bar plot (Figure 4).

**Supplemental Figure Legends**

**Supplemental Table 1. Output from miRDeep2 algorithm and novoMiRank scores for the 103 unique unannotated miRNA candidates.**

**Supplemental Figure 1. Percent GC content of unannotated and annotated miRNAs.** Histogram blot of the percent GC content of the 110 filtered unannotated miRNAs predicted from miRDeep2 and all annotated miRNAs from miRBase v21. Dashed red lines indicate the two standard deviation thresholds from the mean of annotated miRNAs and were used as a filtering criteria.

**Supplemental Figure 2. Predicted targets and their overlaps across applied algorithms. A)** Resulting number of predicted mRNA targets and their overlaps across the three different algorithms applied during target prediction analysis. **B)** Total number of predicted mRNA targets per unannotated miRNA.

**Supplemental Figure 3. Expression of the 38 unannotated miRNA transcripts in tumors.** The expression of the 103 unannotated miRNAs was evaluated in a cohort of 47 liver tumor samples derived from the same patients in which the original miRNA prediction was performed. The expression of 38 miRNAs (39.1% of all the 103 miRNAs discovered) was detected in these tumor samples.

**References**

1. Martinez VD, Vucic EA, Thu KL, Hubaux R, Enfield KS, Pikor LA, Becker-Santos DD, Brown CJ, Lam S, Lam WL: **Unique somatic and malignant expression patterns implicate PIWI-interacting RNAs in cancer-type specific biology**. *Scientific reports* 2015, **5**:10423.

2. Capece V, Garcia Vizcaino JC, Vidal R, Rahman RU, Pena Centeno T, Shomroni O, Suberviola I, Fischer A, Bonn S: **Oasis: online analysis of small RNA deep sequencing data**. *Bioinformatics* 2015, **31**(13):2205-2207.

3. An J, Lai J, Lehman ML, Nelson CC: **miRDeep*: an integrated application tool for miRNA identification from RNA sequencing data**. *Nucleic acids research* 2013, **41**(2):727-737.

4. Gardner PP, Daub J, Tate JG, Nawrocki EP, Kolbe DL, Lindgreen S, Wilkinson AC, Finn RD, Griffiths-Jones S, Eddy SR *et al*: **Rfam: updates to the RNA families database**. *Nucleic acids research* 2009, **37**(Database issue):D136-140.

5. Londin E, Loher P, Telonis AG, Quann K, Clark P, Jing Y, Hatzimichael E, Kirino Y, Honda S, Lally M *et al*: **Analysis of 13 cell types reveals evidence for the expression of numerous novel primate- and tissue-specific microRNAs**. *Proceedings of the National Academy of Sciences of the United States of America* 2015, **112**(10):E1106-1115.

6. McCall MN, Kim MS, Adil M, Patil AH, Lu Y, Mitchell CJ, Leal-Rojas P, Xu J, Kumar M, Dawson VL *et al*: **Toward the human cellular microRNAome**. *Genome research* 2017, **27**(10):1769-1781.

7. Backes C, Meder B, Hart M, Ludwig N, Leidinger P, Vogel B, Galata V, Roth P, Menegatti J, Grasser F *et al*: **Prioritizing and selecting likely novel miRNAs from NGS data**. *Nucleic acids research* 2016, **44**(6):e53.

8. Wake C, Labadorf A, Dumitriu A, Hoss AG, Bregu J, Albrecht KH, DeStefano AL, Myers RH: **Novel microRNA discovery using small RNA sequencing in post-mortem human brain**. *BMC genomics* 2016, **17**(1):776.

9. Backes C, Fehlmann T, Kern F, Kehl T, Lenhof HP, Meese E, Keller A: **miRCarta: a central repository for collecting miRNA candidates**. *Nucleic acids research* 2018, **46**(D1):D160-D167.

10. Fehlmann T, Backes C, Kahraman M, Haas J, Ludwig N, Posch AE, Wurstle ML, Hubenthal M, Franke A, Meder B *et al*: **Web-based NGS data analysis using miRMaster: a large-scale meta-analysis of human miRNAs**. *Nucleic acids research* 2017, **45**(15):8731-8744.

11. Veksler-Lublinsky I, Shemer-Avni Y, Kedem K, Ziv-Ukelson M: **Gene bi-targeting by viral and human miRNAs**. *BMC bioinformatics* 2010, **11**:249.

12. Kertesz M, Iovino N, Unnerstall U, Gaul U, Segal E: **The role of site accessibility in microRNA target recognition**. *Nature genetics* 2007, **39**(10):1278-1284.

13. Kruger J, Rehmsmeier M: **RNAhybrid: microRNA target prediction easy, fast and flexible**. *Nucleic acids research* 2006, **34**(Web Server issue):W451-454.

14. Yu G, Wang LG, Han Y, He QY: **clusterProfiler: an R package for comparing biological themes among gene clusters**. *Omics : a journal of integrative biology* 2012, **16**(5):284-287.

15. Rahmati S, Abovsky M, Pastrello C, Jurisica I: **pathDIP: an annotated resource for known and predicted human gene-pathway associations and pathway enrichment analysis**. *Nucleic acids research* 2017, **45**(D1):D419-D426.
